# Endoscopic Endonasal Surgery for Sinus Fungus Balls: Clinical, Radiological, Histopathological, and Microbiological Analysis of 40 Cases and Review of the Literature

**Published:** 2019-01

**Authors:** Gian-Luca Fadda, Giovanni Succo, Paolo Moretto, Andrea Veltri, Paolo Castelnuovo, Maurizio Bignami, Giovanni Cavallo

**Affiliations:** 1 *Department of Otorhinolaryngology, University of Turin, San Luigi Gonzaga Hospital, Orbassano, Italy.*; 2 *FPO IRCCS, Head & Neck Oncology Unit, Candiolo Cancer Institute, Turin, Italy.*; 3 *Department of Diagnostic Imaging, University of Turin, San Luigi Gonzaga Hospital, Orbassano, Italy.*; 4 *Department of Otorhinolaryngology, University of Insubria, Varese, Italy. *

**Keywords:** Aspergillus, Endoscopic endonasal surgery, Fungal rhinosinusitis, Mycosis, Paranasal sinus fungus ball.

## Abstract

**Introduction:**

Paranasal sinus fungus ball (PSFB) is a non-invasive mycosis, which appears in immunocompetent patients, along with unilateral lesion. The purpose of this study was to analyse various symptoms of PSFB and its radiological, pathological, and microbiological findings. In addition, this study involved the investigation of the incidence of bacterial coinfection and surgical techniques applied for this infection and to report the modern developments in this domain.

**Materials and Methods:**

This retrospective study was carried out on 40 consecutive patients referring for PSFB treatment to the Ear, Nose, and Throat Department in San Luigi Gonzaga University Hospital, Turin, Italy, from April 2014 to 2017. Pertinent literature was reviewed and compared within the specified period. All patients were examined by preoperative computed tomography (CT) scan, and 26 (65%) patients were subjected to magnetic resonance imaging (MRI).

**Results:**

Totally, 33 patients (82.5%) were affected with single sinus infection, whereas most of the cases suffered from maxillary sinusitis. With regard to CT scan findings, microcalcifications were found in 32.5% of the cases; however, mucosal membrane thickening around the fungus ball (FB) was visible in contrast-enhanced CT scans. According to MRI examination, FB showed a characteristic “signal void” on T 2(42.3%). Only 7(17.5%) patients had a positive mycological culture, whereas bacterial coinfections were identified in 47.5% of the cases. Out of 40 patients, 3(7.5%) subjects had only radiological evidence of fungal colonization while having no histopathological evidence. No patient received postoperative antifungal drugs, and there were no serious complications with only one recurrence.

**Conclusion:**

Endoscopic endonasal surgery is the treatment of choice for patients with PSFB receiving no associated local or systemic antifungal therapy. A histopathological study facilitates the confirmation of the diagnosis and exclusion of the invasive form of fungal rhinosinusitis.

## Introduction

Fungal rhinosinusitis (FRS) is a relatively uncommon entity that accounts for 4-13% of chronic rhinosinusitis (CRS) cases (1-3). With regard to the histopathological evidence indicating the presence of fungal hyphae in all anatomical compartments comprising the sinus (4-9), FRS is divided into two categories, namely invasive and non-invasive. Immunodeficient patients are characterized by invasive FRS, whereas the immunocompetent ones are more susceptible to the non-invasive form (10,11). Nowadays, the term ‘fungus ball (FB) of the paranasal sinus (PSFB)’ has replaced the terms ‘mycetoma or aspergilloma’ (4,12,13). Aspergillus fumigatus is reported as the most common pathogenic organism of PSFB (90%), followed by A. niger and A. flavus. However, in the Middle East, *A. flavus *is more common than A*. *fumigatus (14,15).

The FB is regarded as one of the non-invasive FRSs. This condition may rarely affect multiple sinuses; however, it is found in maxillary (80%) and sphenoid sinuses (6,10,13,16-18,19,20). Some of the conditions that may co-exist with FB are bacterial infection and nasal polyps (8,21,22). According to the diagnostic criteria recommended by deShazo et al. (23), preoperative diagnosis of FB necessarily requires the radiological evidence of increased sinus density in the presence or absence of concomitant flocculent calcification (Table.1). 

**Table 1:** Clinicopathological criteria for diagnosis of fungus ball (27)

1. Radiologic evidence of sinus opacification with or without associated flocculent calcifications
2. Mucopurulent, cheesy, or clay-like materials within a sinus
3. A matted, dense conglomeration of hyphae separate from but adjacent to sinus respiratory mucosa
4. A chronic inflammatory response of variable intensity in the mucosa adjacent to fungal elements. This response includes lymphocytes, plasma cells, mast cells, and eosinophils without an eosinophil predominance or a granulomatous response. Allergic mucin is absent on haematoxylin–eosin-stained material
5. No histological evidence of fungal invasion of mucosa, associated blood vessels or underlying bone has been visualized microscopically on Gomori methenamine silver or other special stain for fungus

This should be followed by the implementation of postsurgical histopathology to support the diagnosis (17); however, cultures are often negative (17,24). No specific clinical presentation has been reported for this disease; nevertheless, the incidental finding is possible in entirely asymptomatic patients (4,10,17). Although unilaterality can be regarded as the sole symptom encountered by the clinician (10,11), purulent drainage and oedema can also be considered as the clinical manifestation of this infection. Moreover, endoscopy-guided nasal examination may be helpful in most of the cases (10,11,17).

The gold standard for the treatment of FRS is endoscopic endonasal surgery (EES), which is accompanied with complete fungal debridement, lavage, and reinstatement of the optimal ventilation of the affected sinus (6,11,17,25). Furthermore, this infection has a very low recurrence rate and requires topical or systemic antimycotic therapy (6,11,17,26).In our series of 40 patients with PSFB, we aimed to identify clinical history and preoperative symptoms of PSFB and investigate the nasal endoscopic and reliable radiologic data required for the diagnosis of this pathology before deciding on the therapeutic procedure. In addition, the present study involved the examination of the surgical techniques applied for the management of this infection, its histopathological and microbiological features. Furthermore, anamnestic positivity for receiving dental treatment and anatomical variations accompanying sinonasal FB were investigated. To this end, recent literature on the topic was extensively reviewed.

## Materials and Methods

This study was conducted on 40 patients diagnosed with PSFB and undergoing EES at the Ear, Nose, and Throat Department of San Luigi Gonzaga University Hospital, Turin, Italy, from April 2014 to 2017. The patients with typical radiological and intraoperative findings, as well as histopathological and microbiological examination of fungus hyphae concretions were included in the study. No patient had previously received nasal surgery and those diagnosed with allergic FRS or invasive FRS were excluded from the research. This study was in line with the guidelines of Helsinki Declaration for ethical consideration. Accordingly, written informed consent was obtained from all patients.The enrolled patients were clinically immunocompetent and evaluated preoperatively with nasal endoscopy, paranasal computed tomography (CT), and occasionally with magnetic resonance imaging (MRI). Moreover, the age, gender, and the main complaints of the patients, as well as the location of FB and causative fungus were analysed. No patient was lost during the follow-up period.

**Radiologic examination**

A radiologist and otolaryngologist evaluated the CT scans and MRI findings in order to contribute to the diagnostic process based on the literature review (12,17,20,26-29).The CT results were categorized into three major classes. 

As the first group, the opacification of the involved sinus appeared as an iron-like signal, a metal-dense spot, and an area of “hyperdense material”, also called hyper- attenuating signal with microcalcifications.

 The second category was mucosal membrane thickening around the FB on contrast-enhanced CT scan. 

Marked sinus expansion was considered as the last group. Moreover, with regard to bony wall remodelling, the categorization involved sclerotic or thickened, expanded or thinned, and eroded or dehiscent.

Signal intensity was evaluated using MRI, which was grouped in to three categories: slightly T1-weighted iso-or hypointense, markedly T2-w hypointense (near signal void) and peripheral enhancement of the surrounding mucosa.

The CT scans and MRI findings were also applied to discriminate between the fungal mass and hosting structures in the same patient whenever possible. In addition, the laterality and spreading scope of the disease (i.e., affecting a single sinus or multiple sinuses) were investigated. 

The other variables analysed in the present study included anatomical variations accompanying sinonasal FB, such as a deviated septum, presence of concha bullosa, extent of ostiomeatal unit (OMU) opening, and occurrence of nasal polyps.

**Surgical technique**

All patients underwent an exclusively EES approach under general anaesthesia. Maxillary FB was addressed by opening the sinus through widening its natural ostium. One of the two approaches of transnasal paraseptal and transethmoidal sphenoidotomy was adopted for the management of sphenoid sinus FB depending on the accessibility of sphenoethmoidal recess. In case of using transethmoidal sphenoidotomy due to a protruding rostrum, the transrostral sphenoid approach was chosen, which involved drilling the rostrum to include the natural ostium. 

In all cases, the middle antrostomy and sphenoidotomy were then widened to remove the entire lesion and explore all recesses of the sinus using rigid endoscopes (Karl Storz Endoskope, Germany) with a length of 4 mm and view angles of 45° and 70°. Furthermore, a microdebrider with a curved blade of 40° was used to improve the visualisation of possible residual fungal formations and optimal saline irrigation of the sinus. 

Sinus irrigation was generally carried out at a high pressure, except for those cases in which the CT imaging clearly showed areas of focal bony resorption. Ethmoid localizations were addressed through a complete ethmoidectomy encompassing both the anterior and posterior cells. At the end of the surgical procedure, the nasal cavity was packed, and the packing was removed 48 h after the surgery before discharge.

**Histopathological and microbiological evaluation**

Intrasinus fungi samples were obtained from all subjects and sent for histological and microbiological examination. Moreover, the invasive forms of mycosis had to be ruled out intraoperatively. The mucosal biopsy of the sinus wall was also taken and evaluated histologically. The specimens were homogenised in 0.9% sodium chloride solution, and then subjected to microscopic examination for hematoxylin and eosin stains by such staining techniques as periodic acid-Schiff and Gomori’s methenamine silver. 

Aspergillus, Candida, fungal hyphae, and Mucor species were appeared under different light microscopic assessments. A swab sample was also taken from sinusal purulent secretions and sent to the microbiology laboratory for bacterial culture in both aerobic and anaerobic conditions. 

## Results

**Clinical findings**

Table 2 shows that out of 40 patients diagnosed with PSFB, 29 (72.5%) cases were female. The mean age of the patients was obtained as 52.8 years (age range: 14-84 years). Different symptoms of PSFB included facial pain, purulent nasal discharge, and headache, which were prevalent in 38 (95%), 35 (87.5%), and 30 (75%) patients, respectively. The sphenoid sinus disease was accompanied with headache in all patients. Moreover, concomitant diseases, such as allergic rhinitis, hypertension, gastroesophageal reflux, and type 2 diabetes mellitus, Were Observed in 10(25%), 9(22.5%), 9(22.5%), and (5%) cases, respectively. Furthermore, all patients were immunocompetent and had a negative anamnesis for previous nasal surgery, pulmonary aspergillosis, atopy, or asthma. Purulent secretions were observed in the middle meatus and sphenoethmoidal recess in 16 (40%) and 3 (7.5%) patients, respectively, leading to the preoperative endoscopy in the similar way to that of a bacterial rhinosinusitis. Nevertheless, 18 (45%) subjects had a normal level of purulent secretion. In addition, 3 (7.5%) patients had nasal polyps. There was also a correlation between fungus ball and endodontic treatments in 12 (30%) cases. 

**Table 2:** Clinical symptoms of the paranasal sinus fungus ball according to its location (n=40)

**Involved Sinus**	**No. cases** **(%)**	**Facial pain**	**Purulent rhinorrhea**	**Headache**	**Hyposmia**	**Nasal obstruction**	**Hypogeusia**	**Anosmia**	**Visual disturbance**
*MS	26(65)	26	24	18	21	16	7	5	-
*SS	7(17.5)	6	5	7	3		3		1
MS+SS	5(12.5)	5	5	5	3	4	1		-
MS *(b)	2(5)	1	1	-	-	1	-	1	-
Total(%)	40	38/40 (95%)	35/40 (87.5%)	30/40 (75%)	27/40 (67.5%)	24/40 (60%)	11/40 (27.5%)	6/40 (15%)	1/40 (2.5%)

*MS: maxillary sinus;*SS: sphenoid sinus; *(b): bilateral.

**Radiologic findings**

Table 3 summarizes the characteristics of all 40 patients (100%) undergoing basal CT scan. Both basal and contrast-enhanced CT scans were performed within the same session in 4 (10%) patients. The FB affected a single sinus in 33(82.5%) cases, whereas 7(17.5%) patients had two involved sinuses, namely the maxillary and sphenoid sinuses. No dominant laterality was observed; however, the right and left sides were affected in 20 and 17 patients, respectively, while three cases were affected bilaterally. The FB was found in the maxillary sinus and OMU in 26 (65%) and 19(73%) cases, respectively. In addition, 7(17.5%) patients were affected by sphenoid sinus FB.

**Table 3:** Radiologic characteristics of the paranasal sinus fungus ball in non-contrast computed tomography (n=40) and contrast* computed tomography (n=4).

**Involved Sinus**	**Complete opacity of sinus**	**Partial opacity of sinus**	**Calcifications**	**Iron-like**	**Hyperdense material /** **hyperattenuating**	**Bony sclerosis**	**Marked sinus expansion**	**Mucosal membrane thickening**	*OMU
*MS MS+*SS MS *(b)	28	5	18	9	11	15	5	4	19
*SS	3		3		2	5			
**Total** **(%)**	**31/40** **(77.5%)**	**5/40** **(12.5%)**	**21/40** **(52.5%)**	**9/40** **(22.5%)**	**13/40** **(32.5%)**	**20/40** **(50%)**	**5/40** **(12.5%)**	4* **(10%)**	**19/40** **(47.5%)**

*MS: maxillary sinus;*SS: sphenoid sinus; *(b): bilateral;*OMU: ostiomeatal unit.

The CT scan findings showed a complete or partial opacity in the affected sinus throughout the cohort. The involved sinuses appeared as microcalcifications, hyperdense content, and an iron-like signal in 21(52.5%), 13(32.5%), and 9(22.5%) patients, respectively. Increased thickness of the mucosal membrane was constant on contrast-enhanced CT scans. Thickened or sclerotic bony walls were observed in 20(50%) patients, while thinning or erosion was detected in only 1(2.5%) subject. Septa did not appear to be significantly deviated from the affected sinus. However, 8 (20%) and 11(27.5%) patients had nasal septum deviation and concha bullosa, respectively. Moreover, an associated contralateral rhinosinusitis was observed in 11(27.5%) cases.With regard to the findings of MRI performed on 26 patients, maxillary FB and the localization of sphenoid were found in17(65.4%) and 4(15.4%) cases, respectively. In the remaining 5(19.2%) cases both were affected. Moreover, on the T1-w images, FB was isointense and hypointense in 14(53.8%) and 12(46%) patients, respectively. However, on T2-w images, this lump of fungus was markedly hypointense in 50% (n=13) of the patients and manifested as signal voids in 42.3% (n=11) of the subjects. On T2-w MRI images, the peripheral enhancement of the mucosa surrounding the FB was seen in 7 (26.9%) cases; however, no patient showed extrasinus extension of PSFB. There was also no contrast enhancement in the FB imaging after the administration of gadolinium.

**Histopathological and microbiological analysis**

After EES, the diagnosis of PSFB was confirmed in 37 patients by the histological examination of the surgically removed material and/or a positive fungal culture (Table.4). Histopathology showed a dense aggregation of fungal hyphae with no invasion into the mucosa, blood vessels, or bone of the sinuses.

**Table 4:** Histopathological and microbiological evaluation of paranasal sinus fungus ball (n=40).

**Examination**		**n **	**(%)**
Histological	Aspergillus	31	77.5
	Candida	1	2.5
	Mucormycosis	1	2.5
	Fungal hyphae unable to be differentiated	4	10
	Nasal polyps	3	7.5
			
Culture not made		4	10
Culture negative for fungal growth		33	82.5
Culture positive for fungal growth	Aspergillus fumigatus	4	17.5
	Aspergillus flavus	2	
	Candida	1	
			
Bacterial coinfection	Staphylococcus species (haemolyticus, epidermidis, aureus)	5	47.5
	Pseudomonas aeruginosa	5	
	Streptococcus sp. (intermedius, pneumoniae)	2	
	Haemophilus influenzae	2	
	Other species	5	

According to the histological examinations performed on the specimens obtained from 40 cases, 77.5% (n=31) of the patients were identified with Aspergillus species, followed by Candida species (2.5%, n=1). Moreover, mucormycosis was found in 1 (2.5%) patient; however, fungal hyphae were not differentiated in 4 (10%) patients. Fungal growth cultures were positive in only 7(17.5%) patients, and A. fumigatus was

Identified in four cases.

**Fig 1:**
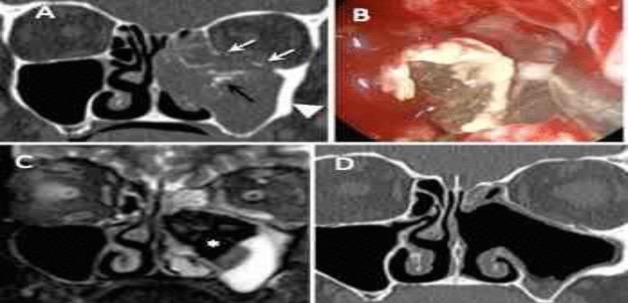
Coronal computed tomographic scans of the paranasal sinuses; A) a heterogeneous opacity of the left maxillary sinus, nasal cavity, and ethmoid air cells with microcalcification (black arrow) and sclerosis of the maxillary sinus wall (white arrowhead); phlogistic erosion of lamina papyracea and orbital floor is also seen (white arrows). Endoscopic removal of a fungus ball in the left maxillary sinus, B) Magnetic resonance imaging shows a signal void on coronal T2-weighted images (small star), C) hyperintense inflamed mucosa at the alveolar recess of the maxillary sinus, D) coronal computed tomography image after surgery showing the complete resolution of fungus ball

The presence of purulent secretions within the sinuses was intraoperatively detected in 19 (47.5%) cases. Such secretions, on which bacterial cultures were performed, appeared similar to those generated by bacterial infections in the same site. Bacterial coinfections were most frequently caused by Staphylococcus species and Pseudomonas aeruginosa. In three cases, the histopathologic results and microbiological cultures were negative for fungal hyphae, while the preoperative radiological imaging (i.e., CT and MRI) and intraoperative endoscopic examination were strongly indicative of FB (Fig.1).

**Postoperative management**

No serious intraoperative or postoperative complication was recorded, and all patients were discharged 2 days after the surgery. Postoperative treatment included nasal saline irrigation twice a day for at least 2 months. In cases with bacterial coinfection (47.5%), systemic antibiotic therapy was administered on the basis of an antibiogram. The topical or systemic antifungal agents were not prescribed. All patients were prospectively followed up through endoscopic monitoring performed within 1-month intervals initiated after the 3^rd^ month of the surgery. Using a rigid endoscope, secretions and crusts were cleaned at the outpatient department. All patients are still in follow-up with a mean duration of 15.8 months, and no relapse has been observed so far.

## Discussion

Certain invasive forms of FRS, which can often be lethal, make this condition a very severe one. The FB is defined as a non-invasive dense aggregation of fungal hyphae in concentric layers usually found in a single sinus cavity (10, 23, 26). The incidence of this condition has apparently been on a growing trend during the past two decades (26). This increased occurrence has been reported to emanate from the massive use of CT and MRI, nasal endoscopy, odontogenic treatment, broad-spectrum antibiotics and steroids, oestrogens, and antitumor therapy (for oncological patients), as well as diabetes mellitus among other metabolic disorders (1, 26,27,30-32). The other factors affecting the incidence of this condition include living in rural areas, a history of undergoing EES, and anatomical variation (33-35). Our results are in line with those reported in the literature (2,12,21,26,33). In the similar articles, the median age of the patients afflicted with this condition was 52.8 years with an evident female predominance (male/female ratio of 0.40). Furthermore, FB (80%) most frequently occurred within the maxillary sinus (2,11,17,21,26,28,33), followed by sphenoidal (5-15%) and ethmoidal (1-15%) sinuses (6,21,26,28,33). However, in the mentioned studies, this condition rarely affected more than one sinus (28).

**Fig2:**
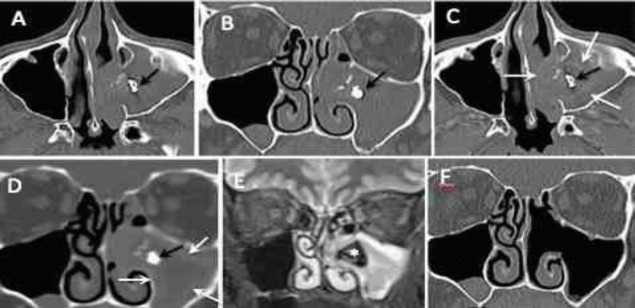
Axial (A) and coronal (B) non-enhanced computed tomography scans; axial (C) and coronal (D) contrast-enhanced computed tomography scans. Complete opacification of left maxillary sinus, nasal cavity, and ethmoid air cells, showing an iron-like density (black arrow) within the maxillary sinus and microcalcifications. There is an associated expansion of the left maxillary sinus. Mucosal membrane thickening around the fungus ball is visible in contrast-enhanced computed tomography scans (C,D) (white arrows). Coronal T2-weighted magnetic resonance imaging (E) with a very low, inhomogeneous signal due to high protein and low water content (small star). Coronal non-enhanced computed tomography scans (F), showing normal air-filled maxillary sinus after surgery

The pathogenesis of PSFB is still unclear. In a number of studies, maxillary sinus FB was reported to correlate with endodontic treatment on the same side of the maxillary bone in over 85% of subjects (26,36-39). In the present study, out of 26 patients having maxillary FB, 12 (46.15%) cases had a previous history of endodontic treatment (Fig.2). Other factors proposed account for the formation of FB are the insufficient drainage of sinusal content due to the impairment of mucociliary transport system (16) and sexual hormones, as indicated by the fact that FB is never found before pubertal development (28,37).

The PSFB presents through nonspecific symptoms (4,17,37). Maxillary FB can present with symptoms mimicking those of a unilateral CRS with unilateral purulent rhinorrhoea (86%), followed by unilateral nasal obstruction, cacosmia, and facial pain (16,26,33). The sphenoid sinus involvement is reported to be most frequently accompanied with headache (86%), followed by purulent nasal discharge in the superior meatus or sphenoethmoidal recess (60-79%) and visual disturbances (up to 20%), such as ptosis, diplopia, or blurred vision (19,26).

 Computed tomography is a reliable diagnostic modality for the diagnosis of PSFB. A non-contrast CT scan will reveal near-to-complete opacification of the involved sinus appearing as a metal-dense spot with microcalcifications or hyperattenuating signal due to the matted fungal hyphae and metabolic deposits (20,28). According to several studies, the prevalence of microcalcifications varies from 33% to 77% (4, 19,27,28,32,36,39).

**Fig 3:**
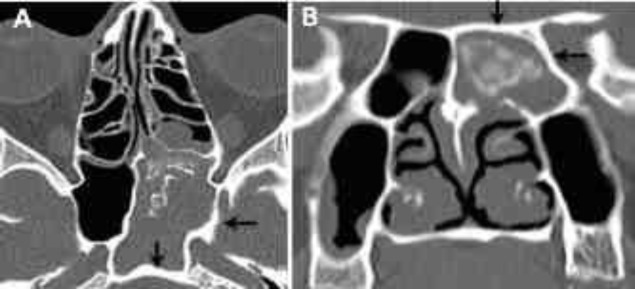
Axial computed tomography scan (A) showing complete opacification of the left sphenoid sinus with associated sclerosis of its bony walls (black arrows), and multiple foci of microcalcifications. On coronal computed tomography scan (B), there is a hyperattenuating rounded mass in the left sphenoid sinus consistent with a  fungus ball

Nicolai et al. (26) reported that the iron-like signal was found more often in maxillary FB than in sphenoid FB (71.9% vs.4.3%), and this difference was significant. Microcalcifications inside the affected sinus and a hyper- attenuating fungal mass were observed in 21 (52.5%) and 13 (32.5%) cases, respectively (Table.3, Fig.3). 

Mucosal membrane thickening around the FB appears to be often hyperattenuating on contrast-enhanced CT scans, whereas basal CT scans do not usually allow for the discrimination of mucosal thickening from a fungal mass (20,28), as also observed in this study. There may be bony wall changes, including sclerosis or thickening secondary to chronic reactive osteitis associated with concurrent bacterial infection (Fig.1a,3a,b,4a and5a). Focal areas of thinning, dehiscence, and marked expansion of the sinus can also present (20, 28). Nicolai et al. (26) found bone erosion in 54.2% and 37.8% of patients with sphenoid and maxillary FB, respectively.

**Fig 4:**
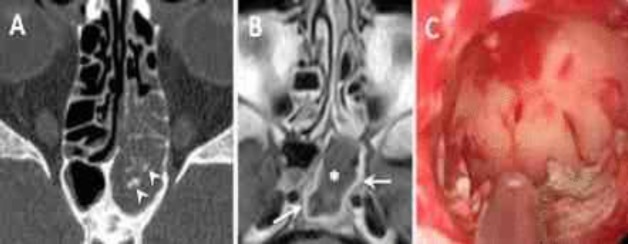
Axial (A) computed tomography scans showing left sphenoethmoidal opacification with associated multiple foci of microcalcifications (white arrowhead). There is also sclerosis of the sphenoidal sinus walls by reactive osteitis. Axial contrast-enhanced T1-weighted magnetic resonance imaging (B) showing thickened, inflamed mucosa at the periphery of mycetoma (white arrows) and intermediate signal of the fungal mass (small star). Intraoperative view of the fungal concretion in the sphenoid sinus floor (C).

An additional MRI with gadolinium may be necessary in order to rule out the suspected invasion of PSFB to the surrounding structures, such as cavernous sinus, intraorbital and intracranial compartments, or remodelled bony walls, on the CT scan and diagnose the clinical symptoms of visual disturbance for sphenoid FB (27).

On MRI images, FB usually appears as an intrasinusal mass that is iso- or hypointense on T1-w and markedly hypointense on T2-w. The lowered signal intensity on T2-w, which can extend as far as to a signal void, is caused both by high iron and manganese concentrations and the presence of calcium under the form of microcalcifications, which have all been noted as the characteristic features of FRS (40,41). The contents of calcium and heavy metals, such as iron, zinc, and manganese, within fungal hyphae most prominently affect the appearance of FB on both CT and MRI imaging (26). For instance, hyperdensity is the natural feature of FB under CT scans. Surgical procedure is one of the therapeutic approaches for PSFB (6,37). The EES technique, facilitating the removal of all fungal debris through a wide opening in the ostium, may be sufficient to reach all therapeutic goals (6,25). This procedure involves the proper ventilation and drainage of sinus without the need for the implementation of the associated local or systemic antifungal therapies (8,10,29,32). 

A number of authors have described the combination of EES with prelacrimal approach through canine fossa. In particular, prelacrimal approach would allow for a thorough clearance of all residual fungal and/or foreign elements given the better visualisation of the anteroinferior recess of the maxillary sinus and lacrimal recess (26). 

Moreover, the anterior wall of the maxillary sinus can be visualised employing angled scopes of 45° and 70° with a combination of an angled suction tube. The repeated high-pressure washing will allow for the clearance of all residual fungal materials eliminating the need for further approaches. Sphenoid FB is a much more serious condition than that the maxillary FB due to its proximity to the brain, optic nerve, and cavernous sinus. There is a broad consensus about the importance of describing the specific involvement of sinuses and implementing sinus wall biopsy (12).

**Fig. 5:**
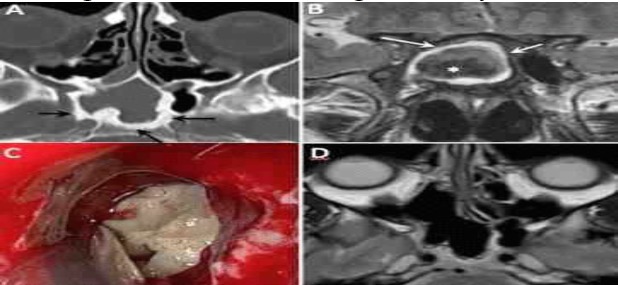
Axial (A) computed tomography scan showing complete opacification and sclerosis of the right sphenoidal walls (reactive osteitis) (black arrows). Coronal T2-weighted magnetic resonance imaging (B) shows isointense signal with associated material filling near signal void (small star). Note also hyperintense signal of thickened inflamed mucosa lining the sinus wall (white arrows). Endoscopic preoperative view of the right sphenoid sinus showing typical friable cheesy yellow to brown material (C). Postoperative axial computed tomography scan showing complete resolution of the right sphenoid disease (D).

A definitive diagnosis of FB ultimately requires both intraoperative endoscopic visual evidence (17) and direct observation of fungal hyphae through histopathological analysis of biopsy samples. Mucosal invasion must also be ruled out (19,28). The macroscopic appearance of FB is a mass reminding of cheese or clay, almost brown in colour, which extends to occupy the sinus (Fig.5). In a study conducted by Pagella et al. (5), it was suggested to take the specimens both from sinus and its mucosa for histological and microbiological analyses. 

Cultures of fungal materials are usually negative or have extremely low sensitivity due to the non-viability of the hyphae inside the mycetoma. In a study performed by Panda et al. (42), the fungal cultures turned out to be positive in 100% of cases; however, this rate was reported as 20-31% in other studies (8,11, 12,17,26,32). 

The patients participated in the current study were all affected by FB, a benign pathology of the paranasal sinuses that did not require postoperative medical therapy. Therefore, direct microscopic examination was considered sufficient without performing polymerase chain reaction amplification and sequencing of the internal transcribed spacer region and calmodulin gene. However, it is believed that it is mandatory to identify fungal species in case of suspecting invasive fungal rhinosinusitis in order to establish an appropriate postoperative drug therapy.

Much interest has been given to the identification of the causative bacteria of CRS. However, concomitant bacterial and fungal coinfection has rarely been explored. Wang et al. (22) analysed 124 cultures of purulent sinus secretions obtained from 123 patients with FB and reported positive results for bacterial coinfections in 91 (73.4%) cases. This finding supports the hypothesis stating that bacterial infections may play a crucial role in the creation of clinical symptoms in a broad number of FB patients. 

Klossek et al. (17) and deShazo et al. (8) advised against the use of antimycotics and considered that surgery alone may be sufficient for the treatment of PSFB as indicated by the low relapse rate reported in several publications. The EES results in a very low recurrence rate with a range of 3.7-7.1%. The relapse of PSFB is the result of the inadequate clearance of fungal concretions (2, 6,11,17,33,37). In the present study, only 17.5% of the patients had a positive fungal culture, and only 1 (2.5%) case had recurrence that needed further sinus surgery.In line with the results obtained by Karthikeyan and Coumare (3), in the present study, it is still unclear why the histopathological and microbiological evidence for fungal disease is negative in some cases, although the radiologic and endoscopic intraoperative findings are strongly indicative of FB. If radiologic and endoscopic intraoperative features are strongly indicative of FRS, immunosupressed and symptomatic patients with high levels of β-D-glucan antigen based on serological testing should undergo antifungal therapy to control infection.

## Conclusion

The clinical presentation and preoperative endoscopic features of PSFB are non-specific. Both CT and MRI can provide a preoperative radiological diagnosis of FRS; however, MRI cannot be performed on all patients due to its high cost. Endoscopic endonasal surgical opening of the infected sinus with the complete removal of the FB is the treatment of choice. A histopathological study confirms the diagnosis and allows the exclusion of the invasive form of FRS. 

However, in some cases, bacterial coinfection may modify the histopathological outcome. If radiologic and endoscopic intraoperative features are strongly indicative of FRS, immunosupressed and symptomatic patients with high levels of β-D-glucan antigen based on serological testing should undergo antifungal therapy to control infection. They should be also closely followed up. On the other hand, if the diagnosis of FB is clear, the recurrence rate is very low and no systemic or topical antifungal therapy is necessary after surgery. 
